# Participatory Development of a Digital Self-Management Intervention for Intensive Care Recovery: Multimethod Study

**DOI:** 10.2196/99235

**Published:** 2026-11-10

**Authors:** Elke Berger, Lisette J W E C van Gemert-Pijnen, Mirjam S van der Veer, Marijke S van der Steen, Dewy Schoonis - Spapens, Daphne Bolman, Diederik A M P J Gommers, Lilian C M Vloet, Margo van Mol

**Affiliations:** 1 Department of Intensive Care Adults, Erasmus MC University Medical Centre, Dr. Molewaterplein 40, Rotterdam, 3015 GD, The Netherlands, 31 0615566554; 2 Psychology, Health & Technology, Faculty of Behavioural, Management and Social Sciences, University of Twente, Enschede, Overijssel, The Netherlands; 3 SGBO [Sociaal Geneeskundige Beroepsopleidingen], Advance training Occupational physician, Radboud University Nijmegen, Nijmegen, Gelderland, The Netherlands; 4 QULI, Amsterdam, North Holland, The Netherlands; 5 Family and Patient Centered Intensive Care (FCIC) & Intensive Care Connect (IC-Connect), Alkmaar, The Netherlands; 6 Research Department of Emergency and Critical Care, Knowledge Centre of Sustainable Healthcare, School of Health Studies, HAN University of Applied Sciences, Nijmegen, Gelderland, The Netherlands

**Keywords:** CeHReS Roadmap, digital self-management intervention, eHealth, intensive care, nurse, recovery, relatives, survivors, Center for eHealth Research and Disease Management

## Abstract

**Background:**

Despite promising developments in eHealth, a critical gap remains in understanding how to design and implement digital self-management interventions that effectively align with users’ needs and the complex context of recovery after treatment in the intensive care unit (ICU).

**Objective:**

This study aimed to develop and iteratively refine a digital self-management intervention for post–intensive care recovery and to explore related user experiences and usability among individuals with lived ICU experience.

**Methods:**

A multimethod participatory development process was used including individuals with lived ICU experience (both survivors and family members) and health care professionals involved in ICU recovery. Between April 2022 and July 2026, guided by the Center for eHealth Research and Disease Management Roadmap consisting of 5 phases, data were collected through inspiration sessions and feedback rounds, individual interviews, usability testing, and pilot use, with qualitative data analyzed thematically and quantitative data analyzed descriptively. Findings from each phase informed the subsequent development steps and were revisited when necessary. The study focused on the specification and iterative refinement of the intervention’s core components based on identified values and contextual requirements, complemented by preliminary indicators of usability and descriptive usage metrics.

**Results:**

Through stakeholder engagement, key functionalities were defined, including structured recovery information, easy 24-7 access, and guidance in navigating care. Two digital self-management modules, the ICU-RecoveryPointer for ICU survivors and the ICU-FamilyCoach for family members, were developed comprising (1) structured information aligned with the different phases of ICU recovery, (2) concise information tiles with layered content for more in-depth background information, (3) patient-reported e-monitoring features, (4) tailored suggestions addressing recovery challenges and emotional processing, (5) an e-diary with a timeline summary function, and (6) a comprehensive content library. During the pilot phase, the ICU-RecoveryPointer reached 33 linked accounts, with 27 users logging in at least once, whereas the ICU-FamilyCoach reached 66 accounts, with 90 monthly unique-user observations. Usage was primarily concentrated on the information tiles, suggesting that users mainly accessed the informational content within the modules.

**Conclusions:**

Two digital self-management modules were designed through a participatory and iterative development process, supporting individuals with lived ICU experience during the recovery trajectory. Preliminary user experiences and usage data provided initial insights into usability and engagement, while also highlighting the need for further evaluation before broader implementation.

## Introduction

Digital technology is rapidly transforming health care, offering opportunities to improve personalized care, treatment accuracy, patient safety, and workflow efficiency [1,2]. Ultimately, the goal is to improve quality of life and support better health outcomes by developing and implementing digital technologies that facilitate value-based health care and improve continuity of care [3,4]. To address this goal, it is increasingly important in today’s world that people are more in charge of their own health care, ranging from preventive strategies and home care to end-of-life situations and shared decision-making in intensive care treatments. Supportive interventions applied through eHealth provide structured technical applications and processes to follow these trends [5,6]. Within the broad field of eHealth, the present study focuses specifically on the development of a digital self-management intervention.

An intensive care unit (ICU) is one of the most high-tech and specialized units in hospitals, providing advanced interprofessional treatment for critically ill patients [7,8]. This group is characterized by multiple morbidities and complex care needs, placing significant demands on accurate prediction and clinical decision-making under conditions of outcome uncertainty [9], which often extend beyond ICU discharge [10,11]. ICU survivors might experience complex recovery needs that necessitate adequate information and supportive care when transitioning to home [12]. Impairments in the physical, cognitive, psychological, and social domains could restrict daily functioning and deleteriously influence quality of life, with mental health playing a predominant role [13,14]. Approximately 50% of ICU survivors develop new or worsening health-related problems across these domains, collectively referred to as post–intensive care syndrome (PICS), which may persist for months or even years [15-17]. The ICU experience may also negatively affect the well-being of family members, ranging from legal representatives to the broader circle of relatives and close friends [18].

Following the uncertain and demanding ICU trajectory, 20% to 40% of family members experience psychological distress, commonly described as PICS-family (PICS-F) [19,20]. These long-term symptoms of PICS and PICS-F are interrelated [14].

Despite a growing awareness of long-term burdens of post-ICU sequelae and the availability of guidelines and recommendations for multimodal rehabilitation approaches [21,22], ICU recovery services remain fragmented, and effectiveness measures remain inconclusive [23-25]. A gap in care, particularly characterized by limited information provision and insufficient communication about the recovery trajectory, has been identified as a shortcoming of current health care systems [26,27]. Moreover, most post-ICU clinics invite (only a selection of) survivors and family members at 2 to 3 months after discharge, thus limiting ongoing professional support [28,29]. These inadequacies highlight the need for accessible and scalable approaches that empower these individuals with lived ICU experience. Digital solutions may offer a means to support self-management and continuity of care throughout the recovery trajectory.

The use of digital technologies and AI in the ICU has the potential to optimize decision-making [30] and resource allocation [31]. Hybrid models that incorporate eHealth, such as telehealth or digital consultations, may help overcome barriers such as low attendance rates for in-person ICU recovery clinics and transportation difficulties among ICU survivors [32]. An innovation with a “digital recovery pathway,” including individualized goal setting, appears promising [33,34]. Other initiatives address a self-guided coping skills training program delivered via a native mobile app platform [35], a virtual exercise class followed by a support group [36], therapist-led partner-assisted cognitive behavioral writing therapy [37], and virtual reality–based intensive psychological interventions [38,39]. Together, these developments demonstrate the potential of digital interventions to support ICU recovery [40]. However, the responsibility for long-term follow-up and coordination of care frequently shifts to primary care, where general practitioners and other primary care professionals may play a central role in monitoring, addressing, and organizing treatment for ongoing health-related impairments [41]. With limited knowledge in the ICU recovery domain, these primary care roles seem insufficiently incorporated into existing health care systems. As a result, many digital ICU recovery initiatives focus on hospital-based support, with limited incorporation of perspectives from stakeholders across the broader care continuum, including primary care and community services.

Despite promising developments in eHealth, a critical gap remains in understanding how to design and implement digital self-management interventions for ICU recovery that adequately align with users’ needs and preferences and the complex context of recovery after treatment in ICU, particularly when professional support is not continuously available.

The aim of this study was to develop and iteratively refine a digital self-management intervention for post–intensive care recovery and to explore related user experiences and usability among individuals with lived ICU experience.

## Methods

### Study Design

A multimethod participatory development process was used to conduct the study with a good fit between technological, human, and contextual factors [42]. This practical approach aligns with the iterative and exploratory nature of early-stage intervention development. To maximize public and patient involvement, representatives of the Dutch Foundation Family and Patient Centered Intensive Care (FCIC), including the patient organization IC Connect, were involved in the research team from the outset and contributed to the study design and conduct of study materials. An a priori protocol has been published [43]. Reporting of the current study followed the Guidance for Reporting Intervention Development Studies in Health Research (GUIDED) [44]. In addition, relevant items from the Guidelines and Checklist for the Reporting on Digital Health Implementations (iCHECK-DH) were addressed to enhance transparency regarding digital health components (Multimedia Appendix 1) [45,46].

No hypothesis testing was performed, as the objective was intervention development rather than effectiveness evaluation. The study focused on the specification and iterative refinement of the intervention’s core components based on identified values and contextual requirements, complemented by preliminary indicators of usability and descriptive usage metrics.

### Study Participation and Recruitment

Study participants comprised 2 participant groups, described as stakeholders and end users. Stakeholders were individuals or representatives who were involved in, affected by, or able to influence the development and implementation of the intervention [47,48], whereas end users were individuals for whom the intervention was intended, in particular ICU survivors and their family members.

#### Stakeholder Representatives

A purposive sampling strategy was used to recruit stakeholders as active collaborators throughout the development of the intervention. Health care professionals involved in ICU recovery nationwide, both within hospital settings and primary care, were identified through the researchers’ professional networks and through snowball sampling. Recruitment continued until at least 1 representative or, where possible, multiple representatives from each relevant discipline were included. These disciplines comprised intensivists, ICU nurses, physiotherapists, speech and language therapists, psychologists, social workers, and general practitioners. In addition, the chairs of Foundation FCIC and IC Connect participated as representatives of individuals with lived ICU experience. Stakeholder roles varied across the different phases of the project according to their expertise, interest, availability, and relevance to the specific development activities. In line with stakeholder theory, stakeholders were mapped in an exploratory manner by the research team (LCMV, MvM, and MSvdS) according to their relative levels of power, legitimacy, and urgency, which together informed their salience within the development process (Multimedia Appendix 2) [49]. To capture a broad range of perspectives, stakeholders were invited to reflect on priorities, identified values, and preliminary design types of the intervention and to contribute to discussions and decision-making during the iterative process.

#### End Users

Adults (aged >18 years) with lived ICU experience (ICU stay>48 hours) were recruited through multiple channels. Depending on the development activity, family members were invited by researchers through telephone contact from 4 hospitals in the southwestern Netherlands or approached during the ICU admission of their loved one. ICU survivors were recruited nationally through peer support groups, the IC Connect newsletter, and social media. No explicit restrictions were applied regarding time since ICU discharge. For pragmatic reasons, only participants who were able to communicate proficiently in Dutch were included. However, participation was recommended only for individuals who self-assessed that they were sufficiently recovered to discuss their ICU experience. No predefined selection criteria were used regarding age, gender, educational level, or cultural background to maximize diversity within the study sample, data collection, and analysis. Family members were eligible if they had been present during the ICU stay and were legally responsible at the time of admission. Bereaved families were excluded.

### Guiding Framework

The Center for eHealth Research and Disease Management (CeHReS) Roadmap was used as the intervention development and implementation framework to guide and structure the development, implementation, and evaluation of the intervention [50]. The road map, established by the Center for eHealth and Wellbeing Research, consists of 5 phases: contextual inquiry, value specification, design, operationalization, and summative evaluation. The phases are connected through formative evaluation circles, representing a process of going back and forth as long as needed in a flexible and agile development approach. Furthermore, the road map facilitated a multidisciplinary team, representing all relevant perspectives (such as target groups including users and health care professionals, designers, technicians, engineers, managers, and researchers) to ensure more diversity in expertise, skills, and knowledge, as well as offering a base for a close-fitting collaboration between users, technology, and context. The road map was adapted pragmatically to fit the context of the study.

### Study Process

Throughout all phases of the development process, between April 2022 and July 2026, multiple interrelated activities were conducted, including desk research, a literature review, surveys, individual and focus group interviews, inspiration sessions, usability testing, and pilot use (Multimedia Appendix 2). The present article primarily reports the activities and findings from the value specification, design, and operationalization phases, which were informed by individual and focus group interviews and inspiration sessions. Researchers acted as process facilitators and methodological leads, coordinating stakeholder engagement and synthesizing the findings into preliminary design considerations. The intervention was developed on an existing web-based eHealth platform, Quli (Quli BV), which provided the underlying technical infrastructure, including secure authentication, content management, and communication functionalities. Rather than developing a new platform, the project focused on designing and iteratively refining the intervention content, navigation, functionality, and user interface within this existing environment. The platform was selected because it was already implemented within the participating health care network and complied with the required privacy and security standards for digital health care applications (Multimedia Appendix 3).

### Data Collection

#### Iterative Refinement

Data collection activities were conducted iteratively using an agile development process, in which findings from each activity informed the subsequent development steps and were revisited when necessary to refine and optimize the digital self-management intervention to fit the complex ICU recovery context [51,52]. Different sources were triangulated and synthesized through researchers’ team discussions, in which emerging insights were compared to enable a comprehensive understanding of the context and implementation of the digital self-management intervention. The preliminary digital self-management intervention was refined based on stakeholder feedback until the design was considered sufficiently mature for pilot testing among end users. Decisions regarding the content, structure, and prioritization of potential elements were made based on a combination of practical considerations and alignment with the technical possibilities. Project governance and security measures are described in Multimedia Appendix 3.

#### Inspiration Sessions and Feedback Rounds

Several inspiration sessions were conducted to explore user values, expectations, and preferred functionalities for digital post-ICU recovery support. The sessions were moderated by MSvdS, DS-S, EB, and MSvdV and included ICU survivors (3 online sessions, total n=22), family members (4 in-person focus group meetings, total n=18), and health care professionals (3 online sessions, total n=35) separately. Using a structured topic guide covering proposed functionalities (Multimedia Appendix 4), participants discussed desirable intervention features and priorities. The identified values and preferred functionalities informed the development of the subsequent survey and the initial design requirements for the intervention.

Interactive feedback rounds were conducted to obtain initial feedback on the usability and perceived fit of the prototype within post-ICU recovery care. The sessions were moderated by MSvdS, DS-S, EB, and MSvdV and involved primary health care professionals (n=3) and family members (n=3). Participants reviewed the prototypes and provided feedback on usability, navigation, content, and alignment with real-world care pathways. The findings were used to refine the prototype prior to subsequent usability testing.

#### Interviews

Individual interviews were conducted to explore health care professionals’ perspectives on the role, content, and added value of a digital self-management intervention for ICU recovery. The interviews were moderated by EB and MSvdV and included ICU nurses (n=13) and intensivists (n=3) involved in ICU follow-up care. A semistructured interview guide was used to explore perceived needs, opportunities, and barriers for digital recovery support (Multimedia Appendix 4). The findings informed the identification of professional values and requirements for the intervention design.

#### Usability Testing

Cognitive walk-throughs were conducted to evaluate the usability, understandability, and ease of navigation of the refined prototype. The sessions were moderated by DS-S, EB, and MSvdV and included ICU survivors (n=10) and family members (n=8). Two participants with mild intellectual disabilities were pragmatically recruited from a group of existing users of a personal health environment for people with Down syndrome. No additional formal eligibility criteria were applied. Both participants were independently living adults who already used a personal health environment in their daily lives. They participated voluntarily and provided informed consent themselves. No adapted consent procedure or additional ethical safeguards were applied, as they were able to provide consent independently. Participants completed predefined tasks using the prototype while being guided by a researcher and recording their observations and feedback in a structured worksheet. The walk-throughs assessed whether the prototype could be used intuitively in realistic recovery situations and whether its content, structure, and navigation supported step-by-step use. The findings informed the final refinement of the prototype prior to pilot implementation.

Task-based usability tests were conducted to evaluate whether the intervention could be used effectively in realistic care scenarios. ICU professionals (n=4) and family members (n=5) completed predefined tasks using the intervention and provided feedback on usability, navigation, and overall user experience. The findings informed the refinements of function requirements before pilot implementation.

#### Pilot Testing

The initial uptake and use of the intervention were explored based on the anonymized usage metrics provided by the platform supplier. These metrics, derived from the platform’s usage logs, included account activation, log-in frequency, and access to the different intervention modules.

The findings from all research activities were synthesized within the successive CeHReS development phases and are therefore presented in the *Results* section according to the stages value specification, design, and operationalization.

### Qualitative Synthesis

Qualitative data obtained from inspiration sessions and feedback rounds were analyzed using a pragmatic qualitative approach. Data from the interviews and usability testing, the file name, date, participant ID, basic demographic information, form and duration of interview, and specifics of the setting were stored in Microsoft Excel format. The audiotapes were transcribed verbatim in TXT format using Atlas.Ti 25 (Lumivero) and stored in the REFI-QDA format. After a check by the principal investigator regarding accuracy, these audiotapes were destroyed. The analysis used a pragmatic, inductive approach focusing on identifying recurring values, needs, experiences, usability issues, and suggestions relevant to intervention development. Findings were coded and organized into conceptually related categories (EB and MSvdV) and were discussed within the research team. The findings were finally synthesized into overarching themes that directly informed subsequent intervention development and prototype refinement.

### Statistical Analysis

For quantitative data in pilot testing, a data dictionary was developed in advance to define the dataset and its variables. Demographic characteristics were not collected, as the intervention was not designed as a data collection tool and users were not asked to complete additional registration forms or questionnaires for research purposes. Indicators of usability and descriptive usage metrics are presented as frequencies and counts. No inferential statistical analyses were performed, as the aim of the study was a descriptive evaluation of usability and usage patterns.

### Rigor and Reflexivity

The moderators of the qualitative meetings (DS-S, EB, MvM, MSvdS, and MSvdV) ensured that all participants had the opportunity to express their views. All moderators had prior training and experience in facilitating qualitative interviews. They used standardized topic guides tailored to the specific aims and target groups. The meetings, which were conducted both online and in person, were documented through field notes and audio recordings to ensure transparency and traceability. Brainstorming and inspiration sessions were summarized in written reports, whereas individual and focus group interviews were transcribed verbatim.

The multidisciplinary research team contributed complementary expertise to uphold methodological rigor. EB, a registered nurse and PhD candidate trained in nursing science, contributed expertise in qualitative data collection and analysis. MSvdV, a registered nurse with experience in coordinating an ICU follow-up clinic, and DB, an individual with lived ICU experience, provided practice-based perspectives. MSvdS, a medical doctor and initiator of an in-person ICU follow-up clinic, served as the overall project lead. DS-S, with a background in digital design, contributed experience in interviewing individuals with low digital literacy and coordinated the inspiration sessions. LCMV, professor of emergency and critical care, provided oversight of the integrity of the study activities and project timelines. MvM, a psychologist and former ICU nurse, contributed expertise in qualitative research in this field and supported the methodological and practical aspects of the study. Reflexivity was supported through regular debriefings and interdisciplinary discussions within the research team, during which interpretations were critically discussed and refined. Furthermore, a quality advisory board, consisting of experts in ICU care, implementation, and eHealth, met on 4 occasions during the project. This board provided independent advice to monitor the study progress, ensure methodological rigor, reflect on interim findings, and safeguard the usability of the digital self-management intervention.

### Ethical Considerations

The Medical Ethics Committee of the Erasmus University Medical Center Rotterdam, the Netherlands, granted approval for the study (MEC-2022‐0153 for the contextual inquiry study and MEC-2022‐0741 for program development and evaluation). The committee evaluated the research proposal and determined that the stipulations outlined in the Dutch Medical Research Involving Human Subjects Act were not applicable to the current research plan. The study was diligently conducted in adherence to the ethical principles delineated in the Declaration of Helsinki (64th World Medical Association General Assembly, Fortaleza, Brazil, October 2013).

Informed consent was obtained from all participants upon enrollment and reviewed verbally before data collection started. They were informed that participation was entirely voluntary and that they could decline to answer any questions or withdraw from the study at any time without providing a reason and without any consequences. No financial compensation was provided for participation. The researchers had no direct therapeutic or clinical relationship with the patients or their relatives. Participants’ emotional boundaries were respected by avoiding overly intrusive or distressing questions. Given the possibility that discussing the ICU admission could evoke distressing memories, professional support from experienced ICU nurses was available during and immediately after the meeting. The coordinating investigator (psychologist and ICU nurse), a clinical psychologist, and an independent ICU clinician were available to provide support and appropriate referral if needed.

## Results

### Value Specification

The value specification phase directed the translation of users’ needs into prioritized values for the digital self-management intervention. The stakeholder group considered the findings from the contextual inquiry (Multimedia Appendix 2) and reached broad agreement on the key values and priorities, which served as input for the specific design requirements of the digital self-management intervention, defining its core elements in terms of content and presentation (Table 1). These values were then translated into tailored content modules, structured signposting for relevant health care professionals, and a user-friendly interface enabling flexible, self-paced use. In addition, presentation requirements emphasized the provision of structured and easily accessible information, guided routes to psychological and social support besides physical recovery management, and features that minimize search burden, while ensuring that the digital intervention complements rather than replaces personal contact with health care professionals.

**Table 1.** Translation of stakeholder values into requirements for a digital self-management intervention for post–intensive care recovery.

	Mapped values and priorities among users	Identified content and presentation requirements
Individuals with lived ICU experience in the Dutch context	*For ICU^a^ survivors* Accessible and clearly structured information Professional guidance and referral transparency Support for physical recovery Support for symptom understanding *For family members* Emotional support and coping assistance Accessible information on post-ICU symptoms Transparency regarding available professional support	Personalized online information Overview of relevant professionals Symptom information videos Rehabilitation exercise videos Emotional distress management support
International perspectives of ICU survivors	Personalized and tailored information provision Access to professional guidance when needed Opportunities for peer connection and shared experiences	Personalized online information Opportunities for e-consultations with health care professionals Digital access to peer support
Priorities of ICU survivors	Positive perception and perceived usefulness of digital support Accessible and inclusive digital design User acceptance of digital health technologies Support for effective and confident digital engagement	Personalized online information
Perspectives of family members	Personalized and tailored information Opportunities for peer support and shared experiences Recognition	Personalized online information Digital access to peer support and narratives
Perspectives of ICU nurses engaged in follow-up clinics	Provision of insights into ICU stay Validation and normalization of recovery needs Guidance in navigating care	Provision of clear, structured information Maintenance of personal connection Ensuring of low-friction usability Embedding of prompts and timing cues

a ICU: intensive care unit.

### Design

#### Prototype Development

The design phase involved the iterative development of a blueprint for the digital self-management intervention, building on insights from the contextual inquiry and the prioritized values translated into concrete content and presentation requirements. The digital self-management intervention was developed and embedded within an existing digital health platform. Stakeholder-identified values were translated into design requirements and subsequently operationalized into concrete features. As an important feature within the design, data generated and stored by users within the platform remain the property of the individual user at all times. Further governance, technical design, and description of the intervention are reported in Multimedia Appendix 3.

The initial concept of the digital self-management intervention, depicted as a patient journey in a storyboard (Figure 1) led to the definition of key telemonitoring functionalities for ICU recovery support (Figure 2).

These early concepts were assessed by ICU survivors (n=22), family members (n=18), and health care professionals (n=35) for perceived usefulness and feasibility, resulting in a design with several tiles representing the preferred specifications (Figure 3).

**Figure 1.**
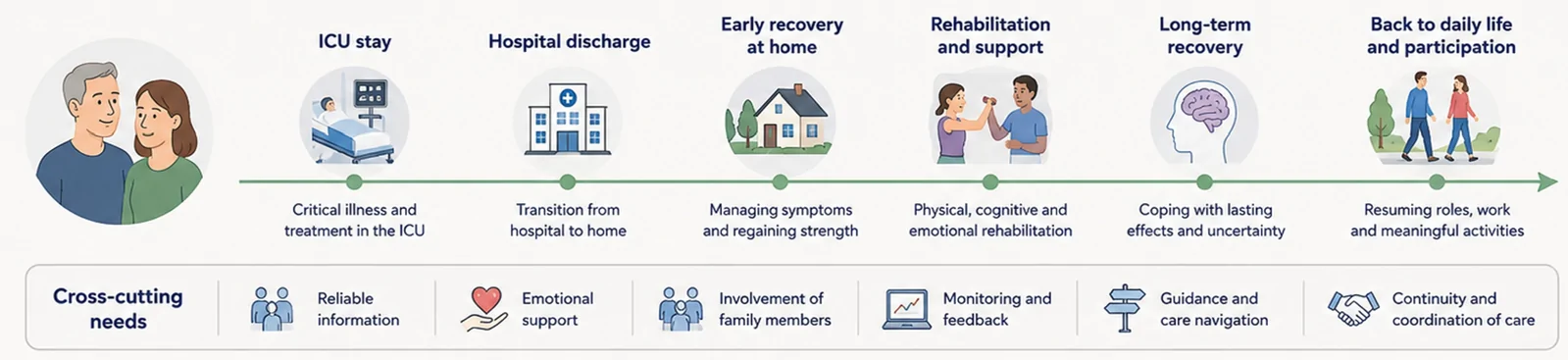
Patient journey storyboard used for value specification during the development of the digital self-management intervention for post-ICU recovery. ICU: intensive care unit.

**Figure 2.**
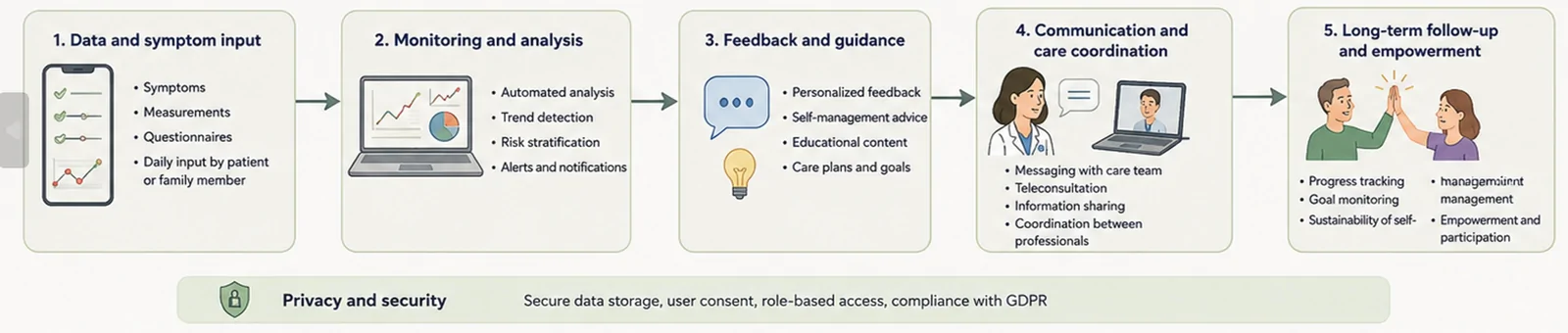
Conceptual storyboard translating user requirements into digital functionalities for the digital self-management intervention for post-ICU recovery. GDPR: General Data Protection Regulation; ICU: intensive care unit.

**Figure 3.**
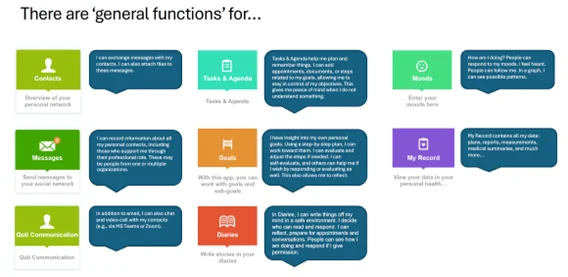
Initial prototype representing informational elements of the digital self-management intervention for post-ICU recovery. The figure illustrates the original text-heavy prototype that participants later perceived as cluttered and difficult to read. ICU: intensive care unit.

However, this design was perceived as too cluttered, unreadable, and visually unattractive among people with lived ICU experience. They reported that it could be improved by further structuring the content according to distinct phases of the recovery process, each including relevant information. Primary care professionals emphasized that the user interface should prioritize clear visualization and intuitive navigation so that users can easily find relevant information and support. Subsequent refinement led to a structured overview with multiple tiles, each providing additional information or a specific functionality, such as an ICU diary or health monitoring (Figure 4).

**Figure 4.**
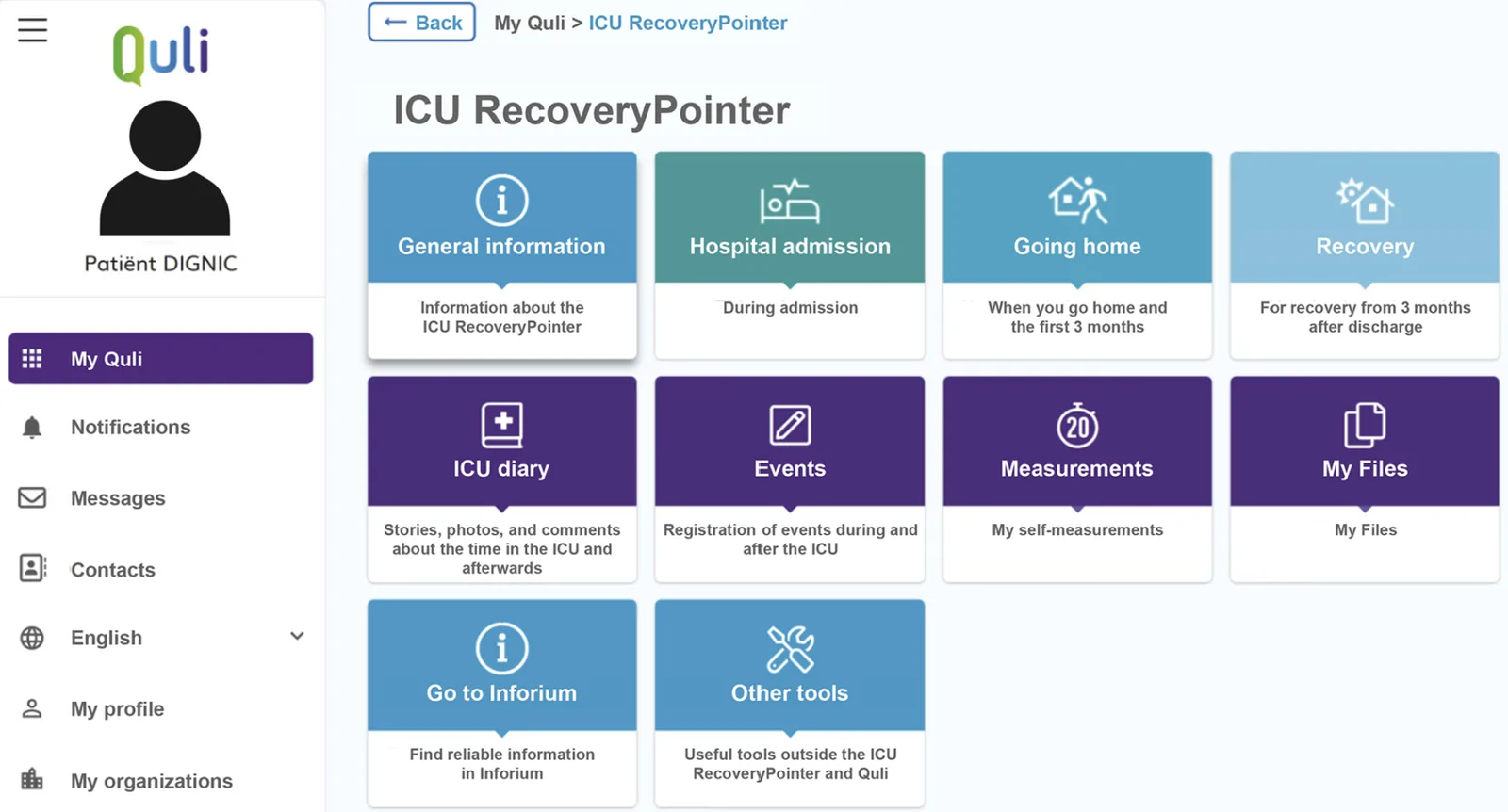
Refined design with tiles representing distinct phases of the recovery process. ICU: intensive care unit.

Three family members confirmed the relevance and completeness of key topics, such as explanations of ICU procedures, common stress reactions, long-term symptoms affecting recovery, and practical guidance for navigating the post-ICU period. They emphasized the need for low-threshold access, layered information, and practical support. Some suggested features, including real-time clinical updates, were considered valuable but not technically or organizationally feasible at this stage. While simplified digital access was considered essential to accommodate varying levels of digital literacy, participants noted a tension between ease of use and the security requirements for handling confidential health data. Participants also stressed the importance of explicitly connecting the intervention with primary care professionals to ensure relevance and facilitate coordination across the ICU recovery trajectory.

Finally, 2 digital modules were developed in a mid-design prototype version: the “ICU-RecoveryPointer” and the “ICU-FamilyCoach” for ICU survivors and family members, respectively. These modules comprised the following core elements: (1) structured information aligned with the different phases of ICU recovery, (2) concise information tiles with layered content for more in-depth background information, (3) patient-reported e-monitoring features, (4) tailored suggestions addressing recovery challenges and emotional processing, (5) an e-diary with a timeline summary function, and (6) a comprehensive content library. A more detailed description and figures of the layered structure are provided in Multimedia Appendix 3.

#### Usability Tests of Prototypes

Prototypes of the intervention, both the ICU-RecoveryPointer and the ICU-FamilyCoach, were prepared for limited real-world use as high-fidelity mock-ups with interaction during inspiration sessions. The full products were used for usability testing with cognitive walk-throughs and field tests.

##### Cognitive Walk-Throughs

Ten ICU survivors tested the ICU-RecoveryPointer and responded positively to the visual design, particularly the use of colors and tile descriptions. They appreciated having all relevant ICU-related information available in one place. However, some participants reported difficulties with complex terminology used in the content. Eight family members tested the ICU-FamilyCoach during their ICU visit. Participants’ comments mainly addressed immediate and practical usability needs relevant in the acute ICU setting, such as intuitive navigation, avoidance of jargon, clearer terminology, the use of visual aids, and direct access to essential information. Their feedback reflected the high cognitive and emotional burden experienced during the ICU phase, with a clear preference for simple, actionable information. Exemplary quotes are presented in Table 2.

**Table 2.** Illustrative quotes reflecting end users’ feedback during the usability testing of the digital self-management intervention for post-ICU^a^ recovery.

Theme	Quote
IC-FamilyCoach home screen	*The colours of the two tiles are very similar. It might be better to use more contrast, especially for users who are colour-blind.* [F3]^b^
General ICU information	*I think it’s a very useful and well-designed tool to gather and look up relevant information. Very well made and meaningful, thank you!* [S1]^c^ *It would be helpful to include a visual map showing the route from the parking area to the hospital entrance, and from there to the ICU.* [F7]
Information about a (digital) ICU diary	*A beautiful concept. It helps make people aware that keeping a diary can be very useful and supportive, also for processing everything afterwards. It is also helpful for sharing with other relatives. Good to have insight into.* [F3]
Information about family participation	*How should children and grandchildren participate? What do you tell them? How old should they be to visit? And when does it become too much for them to see?* [F8]
General opinion at the ICU	*It lacks information about what happens afterward. What is the plan? It might be helpful to include this here as well. It brings a lot of reassurance. And what happens if things don’t go well?* [F8]
Understanding the information	*What a beautiful website. It clearly shows what we’ve missed.* [S5] *Looks good and clear. In the PICS tile, you can click through to information on where to seek or access support.* [F4]
Information about follow-up	*We would have liked to have had this.* [S2] *In the ‘Transfer’ tile, it may be useful to indicate that visiting policies may differ on the ward.* [F4]
Experiences shared by peers	*A video in multiple languages would help many people, and it is also important to include stories from people who had negative experiences.* [F3]
General opinion after the ICU	*Perhaps the ‘Transfer’ tile would be better placed under ‘On the Intensive Care,’ because the transfer process already begins while the patient is still in the ICU.* [F2]
Stress meter/stress self-assessment	*When you are very stressed, it needs to be easier, faster, and simpler. Right now, it’s a lot of information at once.* [F7] *It looks good. I would not use it myself, but I can see that others would find it useful.* [F9]
The advice matches my feelings	*I think it’s a very nice tool that can provide many patients and their families with clarity and support during and long after an ICU admission.* [S7] *These are helpful tips, especially because people often lose themselves when their loved one is in or after the ICU.* [F2]
Opinion about the IC-FamilyCoach	*Very nice, I would be very happy with it. I would definitely use it. It is practical and clear.* [F3]

a ICU: intensive care unit.

b *F*: family member.

c S: ICU survivor.

##### Task-Based Usability Tests

Family members’ (n=5) feedback was reflective and informed by their prior ICU experience. They confirmed the importance of plain-language explanations, support for emotional processing, options to monitor stress over time, and the inclusion of diverse peer experiences. They also pointed to the need for multiple formats, such as photos, simple written prompts, or paper-based materials, to accommodate differences in digital literacy and personal preference. Participants further highlighted aspects that become more salient after the acute phase, such as longer-term coping, meaning-making, and access to broader informational resources.

ICU professionals (n=3) engaged in recovery emphasized that users should be able to determine which information becomes visible and when, depending on their needs during the recovery trajectory. In addition, engagement should start by acknowledging emotions that correspond to the patient’s recovery phase, after which more detailed information and guidance could be provided.

The combined findings of all user tests led to several refinements aimed at improving usability and clarity, including simplified navigation to enhance intuitive use. In addition, content was revised to improve readability and reduce medical terminology, ensuring accessibility for users with varying levels of health literacy. These refinements informed the final prototypes.

### Operationalization

#### Informational Materials

The operationalization phase focused on planning actions for the introduction, dissemination, adoption, and integration of the digital self-management intervention within the intended context. To support this process, materials were developed to inform stakeholders about the purpose, added value, position within health care, and target groups of the ICU-RecoveryPointer and the ICU-FamilyCoach. These materials included short video interviews with individuals with lived ICU experience and health care professionals, in which they shared their perspectives on the new digital self-management intervention. Illustrations were created in line with the visual identity of IC Connect (Figure 5). In addition, a web page was created featuring informative text and images to raise awareness of the intervention. Articles were published in the newsletters of the patient organization IC Connect, the ICU nurses’ association, and the medical ICU association. Furthermore, an official public launch event was organized, involving key stakeholders and representatives from relevant organizations.

**Figure 5.**
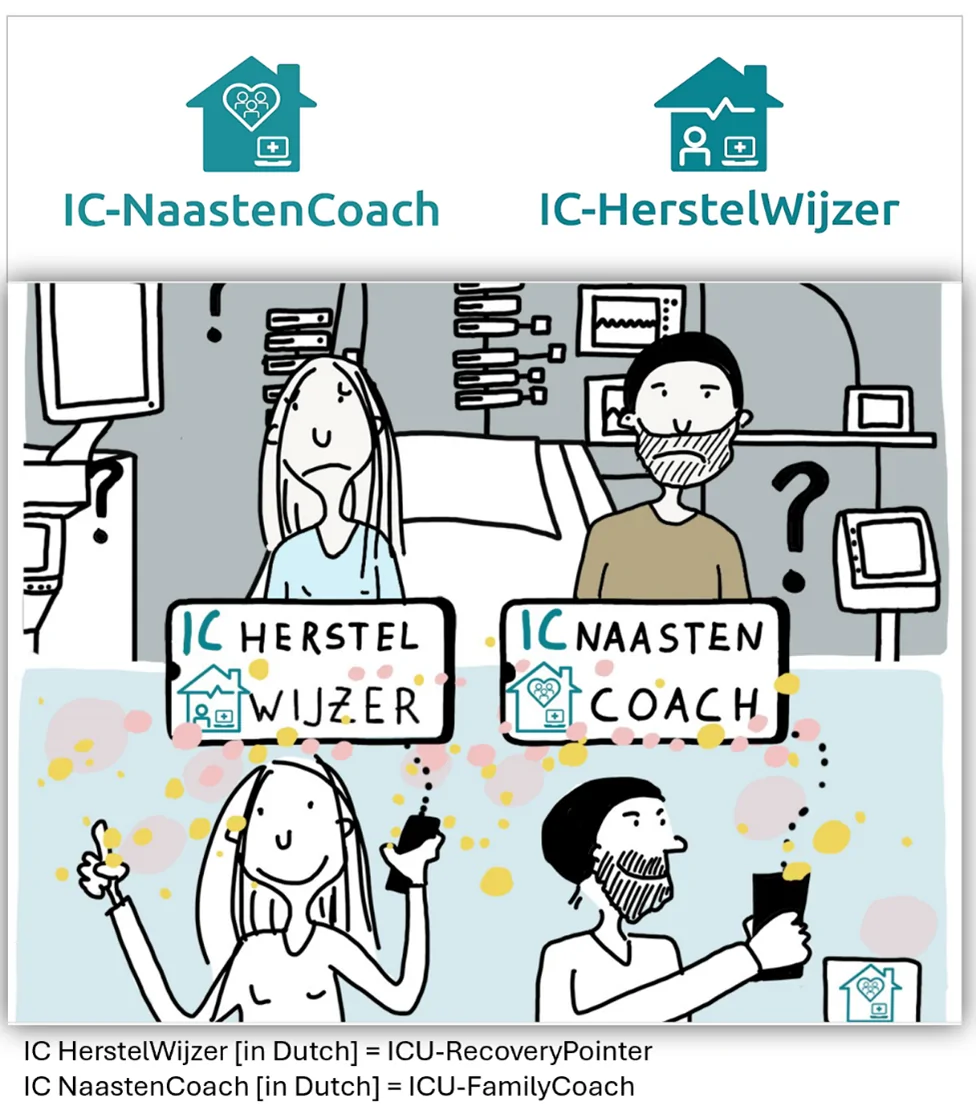
Illustration used to promote awareness of the ICU-RecoveryPointer and ICU-FamilyCoach digital self-management interventions to ICU survivors and family members. ICU: intensive care unit.

The ICU-RecoveryPointer has been publicly and freely available since September 2025, with user accounts provided upon request through IC Connect. The ICU-FamilyCoach has been available since May 2025 in 4 hospitals in the Netherlands, where accounts were provided to participants after informed consent to participate in an evaluation study.

#### User Metrics

The ICU-RecoveryPointer reached 51 end users who received access through linked accounts over the study period (September 2025 to July 2026), of whom 45 were distinct users. Monthly log-in activity ranged from 1 to 14 log-in events. The ICU-FamilyCoach, the user metrics of which are represented in Figure 6, reached 66 end users who received access to the intervention with linked accounts in 8 months (May to December 2025). Across the period, 90 monthly unique-user observations were recorded. Because participants could log in during multiple months, the monthly number of unique users ranged from 4 to 22, accounting for 122 events in total. Usage concentrated on the information tiles (n=565 in ICU-FamilyCoach and n=58 in ICU-RecoveryPointer). To a lesser extent, other actions were established (tile opens: n=45; searches: n=20; data retrievals: n=4). Self-report features were used infrequently (diary entries added: n=13; stress meter viewed: n=15, completed: n=5).

**Figure 6.**
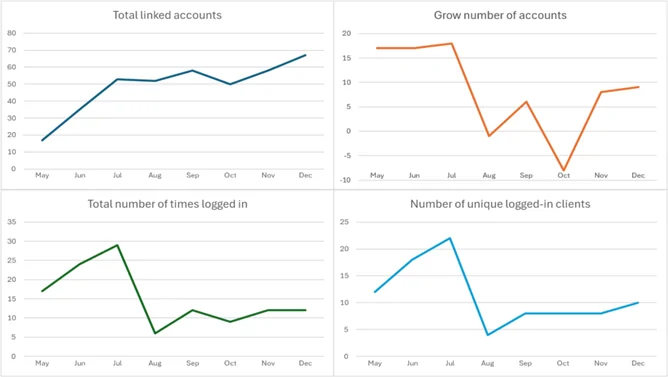
Monthly user metrics of ICU-FamilyCoach during pilot testing, including linked accounts, unique users, log-in events, and information page views.

## Discussion

### Principal Findings

The present article describes the participatory development process and initial design of a digital self-management intervention, specifically the ICU-RecoveryPointer and the ICU-FamilyCoach. These modules were developed to empower individuals with lived ICU experience to regain autonomy after discharge and to tailor their recovery process to their prioritized preferences and needs. Guided by the CeHReS Roadmap, the development trajectory facilitated close collaboration with relevant stakeholders and ensured meaningful public and patient involvement throughout the process.

This study yielded 3 key findings. First, involving ICU survivors, family members, health care professionals, and other stakeholders throughout the development process enabled the identification of user values and contextual requirements that guided intervention design. Second, iterative co-design and usability testing supported the development of a digital intervention that was perceived as relevant and acceptable to its intended users. Third, the pilot provided preliminary insights into user acceptability and engagement, based on descriptive usage metrics and user experiences.

The need for such a digital self-management intervention is underscored by the substantial and persistent burden experienced after ICU discharge and the lack of structured aftercare [53,54]. Similar to a previous study, in the present study, individuals with lived ICU experience emphasized the importance of continuing care, managing expectations, monitoring symptom and recovery progress, and reducing feelings of helplessness as core elements of post-ICU services [55]. Current users’ values addressed timely and tailored information as a key factor in mitigating the symptoms of PICS and PICS-F, consistent with previous research, although informational needs and preferred timing differed between ICU survivors and family members [56]. These findings support the rationale for developing separate, target group–specific modules within our digital self-management intervention.

Although the intervention is designed to support post-ICU recovery, introducing it during ICU admission may enhance continuity of care by familiarizing family members with the platform before discharge. Whether this early introduction improves subsequent uptake and engagement during recovery should be evaluated in future studies [56]. In contrast to many existing online resources [57], the content of our digital self-management intervention was based on clear, accessible lay publications developed by the patient organization IC Connect, making it particularly suitable for individuals with lived ICU experience. Their active involvement throughout the development process, both as a key stakeholder as well as key users, further strengthened the final design. In line with findings from other studies highlighting the added value of engaging individuals with lived ICU experience in innovation and implementation projects [32,58-60], this provides a strong foundation for our study.

eHealth technologies should align with the needs, preferences, routines, and context of all intended users to support adoption and sustained use [6,32,61]. Designing effective interventions for a specific target group therefore requires insight into how individuals think, behave, and make decisions regarding their health and well-being. Using a highly collaborative and methodologically rigorous approach such as the CeHReS Roadmap, the perspectives of health care professionals involved in ICU recovery were actively incorporated into the development process. ICU nurses, in particular, are recognized for their ability to build rapport, understand the burden of post-ICU recovery, facilitate open communication, and demonstrate leadership in value-based health care [4,62]. However, given ongoing ICU nurse shortages and increasing workload pressures, certain supportive tasks may need to be digitized to preserve time for human-centered and relational care. The transformative role of digitization has accelerated the development of digital processes that enhance workflow efficiency, performance, and resource use within nursing practice [63,64]. In this context, innovations such as the 2 digital modules, the ICU-RecoveryPointer and the ICU-FamilyCoach, may contribute to supporting recovery while complementing professional care delivery [65].

eHealth development comprises not only an intervention but also processes for care organization, communication, and knowledge exchange. Therefore, a holistic and participatory development approach is needed [66], in which ICU survivors, family members, nurses, intensivists, physiotherapists, broader recovery teams, primary care professionals, and eHealth designers are actively involved throughout the development process. Understanding the values, goals, and preferences of all stakeholders requires more than acknowledgment; it must be translated into action. The digital self-management intervention was officially launched and featured in several publicly available communication materials. Despite the given attention, such as a social media campaign (ICU-RecoveryPointer) and study-related efforts to introduce the intervention (ICU-FamilyCoach), implementation of the digital self-management intervention appeared to be difficult, given the low numbers in uptake and continuous use. A limitation of the overall development process was that the implementation strategy could have been further strengthened, for example, by more systematically integrating barriers and facilitators identified during earlier phases [50]. Factors that may have influenced the uptake of the intervention include insufficient embedding within a structured care pathway, lack of integration with hospital electronic health records, and the absence of clear ownership or responsibility for a sustainable business model. Furthermore, 2-factor authentication requirements and limited familiarity with digital access to health care may have contributed to resistance in using the intervention. Future research should identify the determinants of successful implementation and evaluate the feasibility, acceptability, and effectiveness of the intervention. If these findings are favorable, broader implementation and long-term sustainability, including the development of an appropriate implementation strategy and business model, should be explored.

The ICU-RecoveryPointer and the ICU-FamilyCoach were designed to accommodate the heterogeneity among the involved participants; however, the composition of a highly engaged and digital enthusiastic participant group may have influenced the value specification and subsequent design requirements. Therefore, the functionality of the 2 modules may have been insufficiently appealing or supportive to keep on track and encourage repeated use among the general target group.

Despite the suggested benefits of digital health care in the literature [40,67], a digital self-management intervention may not fully align with preferences for face-to-face contact [56,60]. A potential barrier to digital support is that its use tends to be lower among certain populations, including older adults, ethnic minorities, individuals with lower socioeconomic status, those with limited health literacy, and people living with chronic illness or disabilities [6]. In contrast, a recent study reported that ICU survivors had sufficient digital health literacy to engage in digital recovery programs [68]. Stakeholders may differ in their backgrounds, interests, and expectations, potentially influencing how the intervention is perceived, adopted, and integrated into practice. A more comprehensive stakeholder analysis could help identify and address these differences to support successful implementation. Although a broad range of stakeholders was included, a formal stakeholder analysis framework was not systematically applied. Rather than comparing perspectives across stakeholder groups, this study aimed to integrate complementary perspectives into a shared set of values and design requirements for the intervention. Nevertheless, these diverse perspectives provided valuable insights that informed the intervention development and laid the foundation for a rigorous participatory design process, consistent with other digital health intervention studies [69]. By addressing informational, emotional, and practical needs, the intervention is promising to offer a scalable approach to complement existing ICU recovery pathways. Future research could further explore differences in perspectives between stakeholder groups during intervention development and implementation. Prior to broader scale-up, a more comprehensive analysis of stakeholders, including policymakers, funding bodies, and governmental organizations, is recommended.

### Limitations

Several limitations should also be acknowledged. First, recruitment of participants through the patient organization and social media may have overrepresented individuals who experienced a particularly difficult recovery trajectory compared with those who recovered more quickly or without long-lasting health problems. This selection bias may have influenced the identification of needs and, consequently, the value specification and design requirements of the digital self-management intervention. Furthermore, recruitment through patient organizations and social media may have preferentially reached individuals who were more motivated or had greater interest in or familiarity with digital health. Consequently, the perspectives of ICU survivors with lower digital literacy or from socioeconomically disadvantaged groups may have been underrepresented, limiting the generalizability of the values and requirements identified during the value specification phase.

Alternatively, this engagement may also have enriched the findings by highlighting opportunities for additional recovery support through digital means. Second, this formative study focused on intervention development and preliminary usage and did not assess health outcomes or clinical efficacy. Consequently, no conclusions can be drawn regarding the intervention’s effects on recovery or other patient- and family-related outcomes. Third, although a broad range of stakeholders was involved throughout the development process, no formal, framework-driven stakeholder analysis was conducted. Consequently, relevant differences in stakeholder influence, interests, and priorities may not have been fully captured. Fourth, no detailed business plan or long-term budget planning was established following the grant application, which may hinder national uptake and sustainability of the intervention. Fifth, the implementation strategy was relatively underdeveloped and initiated late in the overall development process. Although a national digital health strategy exists in the Netherlands, supporting ongoing efforts to expand digitalization in health care, this broader policy context was not sufficiently incorporated during the introduction and dissemination of the ICU-RecoveryPointer and the ICU-FamilyCoach. These challenges underscore the importance of a forthcoming evaluation study, to further explore feasibility, adoption, and contextual determinants of sustained use.

### Conclusion

This study describes the participatory development of a digital self-management intervention to support recovery after intensive care, consisting of the ICU-RecoveryPointer for ICU survivors and the ICU-FamilyCoach for family members. Guided by the CeHReS Roadmap, the intervention was developed through iterative collaboration with individuals with lived ICU experience, health care professionals, and other stakeholders. The resulting modules translate stakeholder needs into accessible digital features aimed at supporting information provision, self-monitoring, and navigation of recovery resources. Preliminary user experiences and usage data provided initial insights into usability and engagement, while also highlighting the need for further evaluation before broader implementation.

## Supplementary material

10.2196/99235 Multimedia Appendix 1 Report of digital health implementation according to the Guidelines and Checklist for the Reporting on Digital Health Implementations (iCHECK-DH).

10.2196/99235 Multimedia Appendix 2 Study overview and contextual inquiry.

10.2196/99235 Multimedia Appendix 3 Governance, technical design, and description of the intervention.

10.2196/99235 Multimedia Appendix 4 Topics for inspiration sessions.

## Abbreviations

FCIC: Foundation Family and Patient Centered Intensive Care
GUIDED: Guidance for reporting intervention development studies in health research
iCHECK-DH: Guidelines and Checklist for the Reporting on Digital Health Implementations
ICU: intensive care unit
PICS: post–intensive care syndrome
PICS-F: post–intensive care syndrome-family

## References

[1] Priyadarshi R, Ranjan R, Sahana BC, Bhandari AK, Kankanala S (2026). A comprehensive overview of smart healthcare technologies in revolutionizing modern rehabilitation practices. Conn Sci.

[2] Ventura-Silva J, Martins MM, Trindade L de L (2024). Artificial intelligence in the organization of nursing care: a scoping review. Nurs Rep.

[3] Stayt LC, Seers K, Tutton E (2015). Patients’ experiences of technology and care in adult intensive care. J Adv Nurs.

[4] Bloomer MJ, Saffer L, Manias E (2024). Important cogs in the wheel: values-based healthcare and what it means for care planning and decision-making in ICU. Intensive Crit Care Nurs.

[5] (2019). Guideline Recommendations on Digital Interventions for Health System Strengthening.

[6] da Silva AA, Merolli M, Fini NA, Granger CL, Gustafson OD, Parry SM (2025). Digital health interventions in adult intensive care and recovery after critical illness to promote survivorship care. J Intensive Care Soc.

[7] Reguera-Carrasco C, Fernandez-García E, Naharro-Álvarez A (2025). Nurse-related complexity of care perceived by critical care nurses: a multicentre qualitative study. Nurs Crit Care.

[8] Trinier R, Liske L, Nenadovic V (2016). Critical care nursing: embedded complex systems. Can J Crit Care Nurs.

[9] Bueno WP, Saurin TA, Wachs P, Kuchenbecker R, Braithwaite J (2019). Coping with complexity in intensive care units: a systematic literature review of improvement interventions. Saf Sci.

[10] Porter LL, Hazeleger L, Bos K (2025). Identifying distinct clusters of ICU survivors by integrating demographic, pre-admission quality of life, and clinical data: a large prospective cohort study. Intensive Care Med.

[11] Liu K, Nakashima T, Goto T (2025). Phenotypes of functional decline or recovery in sepsis ICU survivors: insights from a 1-year follow-up multicenter cohort analysis. Crit Care Med.

[12] Lewis SR, Pritchard MW, Schofield-Robinson OJ, Evans DJ, Alderson P, Smith AF (2018). Information or education interventions for adult intensive care unit (ICU) patients and their carers. Cochrane Database Syst Rev.

[13] Rosa RG, Ferreira GE, Viola TW (2019). Effects of post-ICU follow-up on subject outcomes: a systematic review and meta-analysis. J Crit Care.

[14] Gravante F, Trotta F, Latina S (2024). Quality of life in ICU survivors and their relatives with post-intensive care syndrome: a systematic review. Nurs Crit Care.

[15] Lee M, Kang J, Jeong YJ (2020). Risk factors for post-intensive care syndrome: a systematic review and meta-analysis. Aust Crit Care.

[16] Butcher BW (2026). Post–intensive care syndrome. JAMA.

[17] Ayenew T, Gete M, Gedfew M (2025). Prevalence of Post-intensive care syndrome among intensive care unit-survivors and its association with intensive care unit length of stay: Systematic review and meta-analysis. PLoS One.

[18] Brown C, Hartley P, Forsyth F (2025). What measures have been used to explore the outcomes of family members of critically ill patients: a scoping review. Intensive Care Med.

[19] Putowski Z, Rachfalska N, Majewska K, Megger K, Krzych Ł (2023). Identification of risk factors for post-intensive care syndrome in family members (PICS-F) among adult patients: a systematic review. Anaesthesiol Intensive Ther.

[20] Shirasaki K, Hifumi T, Nakanishi N (2024). Postintensive care syndrome family: A comprehensive review. Acute Med Surg.

[21] Renner C, Jeitziner MM, Albert M (2023). Guideline on multimodal rehabilitation for patients with post-intensive care syndrome. Crit Care.

[22] Federatie-Medisch-Specialisten (2022). Nazorg en revalidatie van intensive care patiënten.

[23] Schofield-Robinson OJ, Lewis SR, Smith AF, McPeake J, Alderson P (2018). Follow-up services for improving long-term outcomes in intensive care unit (ICU) survivors. Cochrane Database Syst Rev.

[24] Leggett N, Emery K, Rollinson TC (2023). Fragmentation of care between intensive and primary care settings and opportunities for improvement. Thorax.

[25] Kean S, Donaghy E, Bancroft A, Clegg G, Rodgers S (2021). Theorising survivorship after intensive care: A systematic review of patient and family experiences. J Clin Nurs.

[26] Schol CMA, Berger E, Rauh SP (2025). Mapping the landscape of post-intensive care recovery interventions and their barriers: A nationwide cross-sectional survey. J Crit Care.

[27] Rea NMA, Neubeck L, Kydonaki K, McHale S (2023). Patients’ experiences and perspectives of post-hospital follow-up care to improve physical recovery for intensive care survivors: A systematic review of qualitative research. Int J Nurs Stud Adv.

[28] Connolly B, Milton-Cole R, Adams C (2021). Recovery, rehabilitation and follow-up services following critical illness: an updated UK national cross-sectional survey and progress report. BMJ Open.

[29] O’Neill B, Green N, Blackwood B (2024). Recovery following discharge from intensive care: What do patients think is helpful and what services are missing?. PLoS One.

[30] Sauer CM, Dam TA, Celi LA (2022). Systematic Review and Comparison of Publicly Available ICU Data Sets-A Decision Guide for Clinicians and Data Scientists. Crit Care Med.

[31] Vernic C, Tamas TP, Petre I, Ursoniu S (2025). Transforming critical care: the digital revolution’s impact on intensive care units. Front Digit Health.

[32] Chatterjee S, Tripathy S, Nayak S, Chakravarty R, Rao PB (2025). Post-intensive care unit clinics: models and implementation - a systematic review. Crit Care.

[33] Rose L, Apps C, Brooks K, Terblanche E, Hart N, Meyer J (2022). Novel digitally enabled care pathway to support postintensive care recovery and goal attainment following critical illness. BMJ Innov.

[34] Rose L, Cox CE (2023). Digital solutions and the future of recovery after critical illness. Curr Opin Crit Care.

[35] Cox CE, Kelleher SA, Parish A (2023). Feasibility of mobile app-based coping skills training for cardiorespiratory failure survivors: the blueprint pilot randomized controlled trial. Ann Am Thorac Soc.

[36] Howroyd F, Earle N, Weblin J (2023). Virtual post-intensive-care rehabilitation for survivors of COVID-19: a service evaluation. Cureus.

[37] Reck J, Gawlytta R, Kesselmeier M (2023). Differential effects of an internet-based cognitive-behavioral writing therapy for reducing PTSD symptoms after intensive care: results of a per-protocol analysis [Article in German]. Psychiatr Prax.

[38] Vlake JH, van Bommel J, Wils EJ (2022). Intensive care unit-specific virtual reality for critically ill patients with COVID-19: multicenter randomized controlled trial. J Med Internet Res.

[39] Drop DLQ, Vlake JH, Wils EJ (2025). Effect of an intensive care unit virtual reality intervention on relatives' mental health distress: a multicenter, randomized controlled trial. Crit Care.

[40] Berger E, Schol C, Meertens-Gunput S (2025). Digital health interventions supporting recovery for intensive care patients and their family members: a scoping review. Mayo Clin Proc Digit Health.

[41] Stewart J, Anderson J, Mayne R (2025). Who is responsible for follow-up after critical illness? GP, ICU and patient perspectives. Crit Care.

[42] Kip H, Keizer J, da Silva MC, Beerlage-de Jong N, Köhle N, Kelders SM (2022). Methods for human-centered eHealth development: narrative scoping review. J Med Internet Res.

[43] van Mol MMC, Kompanje EJO, van Bommel J, Latour JM, ICNaVEN-study group (2023). A study protocol to develop and test an e-health intervention in follow-up service for intensive care survivors’ relatives. Nurs Crit Care.

[44] Duncan E, O’Cathain A, Rousseau N (2020). Guidance for reporting intervention development studies in health research (GUIDED): an evidence-based consensus study. BMJ Open.

[45] Liyanage A, Irfaan S, Moonesinghe L, Kasturiaratchi K, Perrin Franck C, Hussein R (2025). Reporting digital health implementations based on the iCHECK-DH guidelines and checklist: development of an interactive toolkit. J Med Internet Res.

[46] Liyanage A iCHECK-DH: Guidelines and Checklist for the Reporting on Digital Health Implementations.

[47] Van Velsen L, Wentzel J, Van Gemert-Pijnen JE (2013). Designing eHealth that matters via a multidisciplinary requirements development approach. JMIR Res Protoc.

[48] Panteli N, Pitsillides B, Pitsillides A, Samaras G (2010). Web Mobile-Based Applications for Healthcare Management.

[49] Mitchell RK, Agle BR, Wood DJ (1997). Toward a theory of stakeholder identification and salience: defining the principle of who and what really counts. Acad Manag Rev.

[50] Kip H, Beerlage-de Jong N, van Gemert-Pijnen LJEWC, Kelders SM (2025). The CeHRes Roadmap 2.0: update of a holistic framework for development, implementation, and evaluation of eHealth technologies. J Med Internet Res.

[51] Wang S, Hanneman P, Xu C (2020). Critical Care Recovery Center: a model of agile implementation in intensive care unit (ICU) survivors. Int Psychogeriatr.

[52] Lindroth H, Sahajwani J, Hudson M (2025). Applying an agile science roadmap to integrate and evaluate ethical frameworks throughout the lifecycle and use of artificial intelligence tools in the intensive care unit. Crit Care Nurs Clin North Am.

[53] Herridge MS, Azoulay É (2023). Outcomes after critical illness. N Engl J Med.

[54] Hermann M, Karner V, Schaller SJ (2026). Update on long-term physical disability after critical illness: the burden of post-intensive care syndrome. Intensive Care Med.

[55] McPeake J, Boehm LM, Hibbert E (2020). Key components of ICU recovery programs: what did patients report provided benefit?. Crit Care Explor.

[56] Sevin CM, Boehm LM, Hibbert E (2021). Optimizing critical illness recovery: perspectives and solutions from the caregivers of ICU survivors. Crit Care Explor.

[57] Hanci V, Otlu B, Biyikoğlu AS (2024). Assessment of the readability of the online patient education materials of intensive and critical care societies. Crit Care Med.

[58] Skovlund PC, Nielsen BK, Thaysen HV (2020). The impact of patient involvement in research: a case study of the planning, conduct and dissemination of a clinical, controlled trial. Res Involv Engagem.

[59] Watland S, Solberg Nes L, Hanson E, Ekstedt M, Stenberg U, Børøsund E (2023). The Caregiver Pathway, a model for the systematic and individualized follow-up of family caregivers at intensive care units: development study. JMIR Form Res.

[60] Haines KJ, Leggett N, Hibbert E (2022). Patient and caregiver-derived health service improvements for better critical care recovery. Crit Care Med.

[61] Klaic M, Kapp S, Hudson P (2022). Implementability of healthcare interventions: an overview of reviews and development of a conceptual framework. Implement Sci.

[62] Rousseau AF, Fraipont V, Preiser JC (2025). Post-intensive care syndrome is a potential consequence of critical care: can intensivists afford to look away?. Intensive Care Med.

[63] Robert N (2019). How artificial intelligence is changing nursing. Nurs Manage.

[64] Ross A, McGrow K, Zhi D, Rasmy L (2024). Foundation models, generative AI, and large language models: essentials for nursing. Comput Inform Nurs.

[65] Yilmaztürk N, Kose İ, Cece S (2023). The effect of digitalization of nursing forms in ICUs on time and cost. BMC Nurs.

[66] Lai DJ, Liu Z, Johnston E, Dikomitis L, D’Oliveira T, Shergill S (2024). Exploring the effectiveness of eHealth interventions in treating Post Intensive Care Syndrome (PICS) outcomes: a systematic review. Crit Care.

[67] Leggett N, Abdelhamid YA, Daniel NE (2026). Digital health literacy of ICU survivors: a prospective cohort study. J Crit Care.

[68] Johnson GU, Robert G, Lee CS, Boehm L, Pandharipande PP, Ely EW (2026). Reimagining intensive care unit recovery: the Critical Illness Survivorship Programme for humanising post-intensive care unit care - a discussion paper. Int J Nurs Stud.

[69] Twamley J, Monks R, Beaver K (2023). Using experience-based co-design to prioritise areas for improvement for patients recovering from critical illness. Intensive Crit Care Nurs.

[70] van Mol M (2026). Intensive care follow-up service: developing and implementing a nurse-led e-Health intervention for relatives of intensive care survivors. DataverseNL.

