# Supplementary material for: Participatory Development of a Digital Self-Management Intervention for Intensive Care Recovery: Multimethod Study
Source: JMIR Form Res. 2026 Nov 10;10:e99235. doi: 10.2196/99235 (PMC13750520; doi:10.2196/99235)
Supplement: Supplementary file 1 [file formative-v10-e99235-s001.docx]

Multimedia Appendix 1

Report of digital health implementation according to the iCHECK-DH Guideline

Title

Participatory Development of a Digital

Self-Management Intervention for Intensive Care Recovery: A Multimethod Study

Reporting of this study followed the *Guidance for Reporting Intervention Development Studies in Health Research* (GUIDED), as the primary aim of this article was to describe the participatory development process and initial design of a digital self-management intervention. Given the digital nature of the intervention, relevant items from the *iCHECK-DH Guidelines and Checklist for Digital Health Implementations* were additionally addressed to ensure transparency regarding data governance, privacy, cybersecurity, and technical infrastructure. As the present article focuses on intervention development rather than full-scale implementation or effectiveness evaluation, certain implementation-specific iCHECK-DH items were not applicable.

Abstract

**Background:** Despite promising developments in eHealth, a critical gap remains in understanding how to design and implement digital self-management interventions that effectively align with users’ needs and the complex context of recovery after treatment in the intensive care unit (ICU).

**Objective:** The objective of this study was to develop and iteratively refine a digital self-management intervention for post-intensive care recovery and to explore its usability and user experiences among individuals with lived ICU experience.

**Methods:** A multimethod participatory development process was used including individuals with lived ICU experience (both survivors and family members) and healthcare professionals involved in ICU recovery. Between April 2022 and February 2026, and guided by the Center for eHealth Research and Disease Management Roadmap consisting of five phases, data were collected through inspirational sessions and feedback rounds, individual interviews, usability testing, and pilot use, with qualitative data analysed thematically and quantitative data descriptively. Findings from each phase informing subsequent development steps and were revisited when necessary. The primary outcomes were the specification and refinement of core components to build a digital self-management intervention aligned with stakeholder-identified values and contextual requirements. Secondary outcomes included preliminary indicators of usability and descriptive usage metrics.

**Results:** Through iterative stakeholder engagement, key functionalities were defined, including structured recovery information, easy 24/7 access, and guidance in navigating care. Two digital self-management modules, the ICU-RecoveryPointer for ICU survivors and the ICU-FamilyCoach for family members, were developed comprising (1) structured information aligned with the different phases of ICU recovery; (2) concise information tiles with layered content for more in-depth background information; (3) patient-reported e-monitoring features; (4) tailored suggestions addressing recovery challenges and emotional processing; (5) an e-diary with a timeline summary function; and (6) a comprehensive content library.

**Conclusions:** Through a participatory and iterative development process, this study resulted in two digital self-management modules designed to support individuals with lived ICU experience and their family members during the recovery trajectory. By addressing informational, emotional, and practical support needs, the intervention offers a scalable approach to complement existing ICU follow-up services.

Introduction

Item 3 The context

The study setting was both regionally through ICU follow-up services of four hospitals in the South Western part of the Netherlands and nationally through public media.

Despite a growing awareness of this long-term burden of post-ICU sequelae, and the availability of guidelines and recommendations for multimodal rehabilitation approaches (underpinned with a national guideline), ICU recovery services remain fragmented and effectiveness measures inconclusive.

The context inquiry is one of the phases described in Multimedia Appendix 2, although not specified to facilitators and barriers regarding the digital self-management intervention.

In the limitations has been described that a national digital health strategy exists in the Netherlands, supporting ongoing efforts to expand digitalization in healthcare, although this broader policy context was not sufficiently incorporated during the introduction and dissemination of the intervention.

Item 4. The problem statement

Despite promising developments, little is known about how to design and implement eHealth, such as digital self-management interventions for ICU recovery, that adequately align with users’ needs, preferences, and the context of post-ICU recovery, particularly when professional support is not continuously available. Therefore, participatory and user-centred development approaches may offer valuable guidance for the design of a digital self-management intervention that aligns with ICU recovery contexts.

A health system challenge seems not applicable in this phase and context.

Item 5 Similar interventions

Fully reported in the introduction section.

Methods

Item 6 Aims and objectives

To develop and iteratively refine a digital self-management intervention for post-intensive care recovery and to explore its usability and user experiences among individuals with lived ICU experience.

No hypothesis testing was performed, as the objective was intervention development rather than effectiveness evaluation. The primary outcomes were the specification and iterative refinement of the intervention's core components based on identified values and contextual requirements. Secondary outcomes included preliminary indicators of usability and descriptive usage metrics.

Item 7 Blueprint summary

Two digital modules were developed: the ‘ICU-RecoveryPointer’ and the ‘ICU-FamilyCoach’ for ICU survivors and family members respectively. These modules comprised the following core elements: (1) structured information aligned with the different phases of ICU recovery; (2) concise information tiles with layered content for more in-depth background information; (3) patient-reported e-monitoring features; (4) tailored suggestions addressing recovery challenges and emotional processing; (5) an e-diary with a timeline summary function; and (6) a comprehensive content library.

Multimedia Appendix 3 is a detailed description of the developed digital self-management intervention.

Item 8 Technical design

The technical infrastructure, including hardware and software components, was implemented within an existing digital platform. Further details are described under subheading ‘Design’ in the results section and in Multimedia Appendix 3.

Item 9 Target

A purposive sampling strategy, including the use of the researchers’ professional networks and snowball sampling techniques, was employed to recruit healthcare professionals involved in ICU recovery, either within hospital settings or in primary care.

A convenience sample of adults (>18 years) with lived ICU experience (ICU stay >48 hours), either as ICU survivors or family members, was recruited.

Item 10 Data

This is extensively described in the Methods section, under data collection and analysis. Project governance, including privacy and security measures are described in Multimedia Appendix 3. Data were managed according to the FAIR principles, ensuring findability, accessibility, interoperability, and reusability, and were securely stored in compliance with institutional and legal standards.

**Legal framework and compliance**

This project complied with the European General Data Protection Regulation (GDPR; EU 2016/679), the Dutch Implementation Act of the GDPR, and the Dutch Code of Conduct for Health Research. Data governance procedures were reviewed in accordance with institutional policies for secure data handling. Roles, responsibilities, and intellectual property rights were formalized in a consortium agreement between collaborating partners.

**Data ownership and governance structure**

All study data are owned by and stored under the responsibility of the principal investigator’s affiliated institution. Access to data is restricted to authorized research team members based on role-specific permissions. Participants do not have direct access to the research database. However, in accordance with GDPR provisions, participants may request access to their personal data. Following identity verification, an extract of the individual’s data is securely transferred using encrypted file-sharing services (SURFfilesender).

**Data life cycle management**

*Data collection*

Data collection activities were conducted iteratively using an agile development process, in which findings from each activity informed subsequent development steps and were revisited when necessary to refine and optimize the digital self-management intervention to fit the complex ICU recovery context.

*Data processing and modification*

Qualitative data obtained from inspiration sessions and feedback rounds were analysed using a pragmatic qualitative approach. Researchers independently reviewed the notes and transcripts to identify recurring topics, user needs, usability issues, and design suggestions. For qualitative data from the interviews and usability testing, the file name, date, participantID, basic demographic information, form and duration of interview, and specifics of the setting were stored in Excell format. The audiotapes were transcribed verbatim in txt-format using AtlasTi© software and stored in REFI-QDA-format. For quantitative data in pilot testing a data dictionary was developed in advance to define the dataset and its variables. No demographic characteristics were collected. Indicators of usability and descriptive usage metrics are presented as frequencies and counts.

*Data storage and retention*

All research data were pseudonymized and stored on secure institutional servers located in the Netherlands. Audio recordings were deleted after verification of transcription accuracy. Data retention follows institutional and legal retention policies.

*Data sharing and suppression*

Only de-identified datasets may be shared upon reasonable request and in compliance with legal and institutional regulations. Metadata are publicly available (doi:10.34894/PMB1B5). No directly identifiable personal data are shared.

*Data protection and cybersecurity*

Data protection measures were implemented according to principles of privacy by design and security by design. Access to systems was secured through authentication procedures and role-based authorization. Data transfer was encrypted, and all systems complied with institutional cybersecurity standards.

In the event of a suspected data breach, institutional incident response procedures are followed, including immediate notification of the designated Privacy Officer, risk assessment, documentation, and, if required, notification of relevant supervisory authorities.

*Data integration and interoperability*

The intervention was designed as a stand-alone digital application with limited integration into existing electronic health record systems during the development phase. Interoperability considerations included the use of standardized, machine-readable formats (e.g., XML) to facilitate potential future integration.

*Cross-border data transfer*

No cross-border data transfer agreements were required, as all data were collected, processed, and stored within the Netherlands.

Item 11 Interoperability

This is described in Multimedia Appendix 3.

Item 12 Participating entities

The parties involved in the development and ownership are described in Multimedia Appendix 3, while funding has been acknowledged at the end of the main document.

Item 13 Budget planning

We did not report on budget planning. This is described as a limitation of the study.

Item 14 Sustainability

We did not report on sustainability as we have no data or plan for that.

Results

The coverage is intended national.

The results of all the phases in the CeHReS roadmap have been described.

In the discussion section we have elaborated on the value, the success of development and the pitfalls in implementation.

Item 15-18, implementation, are not applicable in current study.

Discussion

Item 19 Discussion and conclusions

For the discussion, see the main document.

The conclusion is as follows: This study describes the participatory development of a digital self-management intervention to support recovery after intensive care, consisting of the ICU-RecoveryPointer for ICU survivors and the ICU-FamilyCoach for family members. Guided by the CeHReS roadmap, the intervention was developed through iterative collaboration with individuals with lived ICU experience, healthcare professionals, and other stakeholders. The resulting modules translate stakeholder needs into accessible digital features aimed at supporting information provision, self-monitoring, and navigation of recovery resources. The self-management intervention could enhance autonomy and getting back a feeling of control among individuals with lived ICU experience. Future research should evaluate the feasibility, adoption, and long-term impact of the intervention in routine care.

General

Because implementation is not the main part of this study, we cannot provide all the mandatory items.
