# Supplementary material for: Participatory Development of a Digital Self-Management Intervention for Intensive Care Recovery: Multimethod Study
Source: JMIR Form Res. 2026 Nov 10;10:e99235. doi: 10.2196/99235 (PMC13750520; doi:10.2196/99235)
Supplement: Supplementary file 2 [file formative-v10-e99235-s002.docx]

Multimedia Appendix 2

Study overview and Contextual Inquiry

The Center for eHealth Research and Disease Management (CeHReS) Roadmap was used as the theoretical framework to guide and structure the development, implementation, and evaluation of the intervention. Throughout all phases of the development process, between April 2022 and July 2026, multiple interrelated activities were conducted, including desk research, a literature review, surveys, individual and focus group interviews, inspiration sessions, usability testing, and pilot use (Table 1). Here we report the main findings from the Contextual Inquiry.

Table 1. Details in study process and data collection

|  | **What** | **How** | **Number**  (if applicable) | **Outcome** |
| --- | --- | --- | --- | --- |
| *Contextual Inquiry; to assess users’ needs and priorities, identify relevant stakeholders, analyse the current situation, and explore main points for improvement*  *Timeline April 2022 – September 2023* | | | | |
|  | Desk research | Exploring general reports, patient driven research agenda, comments from lay websites and social media. |  | Research gap identification. |
|  | Stakeholder identification | Using expert recommendation and snowball sampling. |  | Stakeholder map. |
|  | Brainstorm session | Organizing roundtable sessions that included group discussions and other interactive formats  (moderated by MvM and LV). | 1 online workshop followed by a two-day in-person meeting | Animas and client journeys of an ICU survivor and a family member. |
|  | Focus group meeting | Using patient journey mapping, input was gathered from primary care professionals regarding a potential digital self-management intervention (moderated by MvdS and DS). | n = 4 key informants including general practitioners and physiotherapists | Deeper insights, suggestions and advices. |
|  | Individual interviews | Ensuring broad societal alignment across religious and cultural backgrounds, low literacy and health literacy levels (MvM). | n = 4 key informants from specific communities | Deeper insights, suggestions and advices. |
|  | Individual interviews | Validating and further exploring the needs of individuals with lived ICU experience from non-Dutch backgrounds  (supervised by MvM). | n = 12 | Deeper insights, suggestions and advices. |
|  | Survey | Identifying priorities for digital post-ICU recovery, both in the Dutch context and in comparison with three European countries. | n = 227 participants in the Dutch context  n = 106 participants in European comparison | Recognized values for individuals with lived ICU experience. |
|  | Literature review | Systematically approaching literature for review on existing eHealth applications in ICU recovery pathways. |  | Overview and literature summary. |
| *Value specification; to formulate and prioritize stakeholder-identified values for translation into design requirements and subsequently operationalized into concrete features for the eHealth application.*  *Timeline September 2022 – December 2023* | | | | |
|  | Inspirational sessions | Exploring user values in digital post-ICU recovery (moderated by MvdS, DW, EB and MvdV). | n = 22 ICU survivors  n = 18 family members  n = 35 professionals | Identified values used for survey development. |
|  | Individual interviews | Exploring opinions and ideas regarding a digital self-management intervention in ICU recovery  (moderated by EB and MvdV). | n = 13 ICU nurses and 3 intensivists involved in follow-up clinics | Recognized values for ICU professionals engaged in recovery. |
| *Design; to create the actual eHealth application with prototypes and visual representations.*  *Timeline September 2023 – December 2024* | | | | |
|  | Interactive feedback rounds | Inspecting usability at first sight (moderated by MvdS, DS, EB and MvdV) | n = 3 primary healthcare professionals  n = 3 family members | Feedback for refinement of prototype. |
|  | Cognitive walkthroughs | Exploring usability and ease of navigation  (controlled by DS, EB and MvdV). | n = 10 ICU survivors  n = 2 persons with intellectual disability  n = 8 family members | Feedback for refinement of prototype. |
|  | Task-based usability tests | Evaluating the intervention in a real-world setting | n = 4 ICU professionals  n = 5 family members | Final product |
| *Operationalization; to plan actions for the introduction, dissemination, adoption, and internalization of (a first functioning version of) the eHealth application* *in the intended context.*  *Timeline April 2024 – July 2026, with official first public launce in June 2024* | | | | |
|  | Presentations | Introducing the intervention publicly to provide potential users with information |  | Communication and implementation plan |
|  | Pilot test | Preliminary user metrics of the intervention |  |  |
| *Summative evaluation; to analyse the impact of the eHealth application.*  *Timeline January 2025 - July 2026* | | | | |

**Contextual inquiry**

The contextual inquiry served to provide initial insight into the needs, scope, desired functionalities, and contextual requirements of the digital self-management intervention among people with a lived ICU experience. Through consultations with representatives from patient organisation IC Connect and desk research including a patient journey and patient driven research agenda [1], several contextual challenges in post-ICU recovery were identified. These challenges included fragmented follow-up care, limited accessibility of recovery information outside scheduled appointments, and uncertainty regarding appropriate referral pathways. Findings highlighted a users’ need for accessible, continuous, and user-centered support beyond traditional post-ICU recovery.

Relevant stakeholders were identified through expert recommendations from the research team. In line with stakeholder theory, stakeholders were mapped in an exploratory manner by the research team (LV, MvM, MvdS) according to their relative levels of power, legitimacy, and urgency, which together informed their salience within the development process (Table 2). The aim of stakeholder engagement was to identify and synthesize complementary perspectives to inform intervention development rather than to compare views between stakeholder groups. This phase-specific mapping of stakeholders reflected the iterative and practical development approach. They were actively invited to reflect on and contribute to value specification, and to participate in discussions and decision-making processes related to the design, for example during inspiration sessions and other interactive development activities.

Table 2. Stakeholder map

|  |  | **Role(s)** | **Involved** | **Interest** | **Influence** |
| --- | --- | --- | --- | --- | --- |
| Individuals with lived ICU experience | | |  |  |  |
|  | ICU survivors | End-users | - Consulted - Co-designed - Tested prototypes - Advised / reviewed | High | High |
|  | Family members | End-users | - Consulted - Co-designed - Tested prototypes - Advised / reviewed | High | High |
| Healthcare professionals | | |  |  |  |
|  | Intensivist | End-users  Implementers | - Consulted - Implementation partner | Low | High |
|  | ICU nurses | End-users  Implementers | - Consulted - Implementation partner | High | Neutral |
|  | General practitioner | End-users | - Consulted | Neutral | Low |
|  | Physiotherapist | End-users | - Consulted | Neutral | Low |
|  | Language and speech therapists, psychologist, social worker, rehabilitation physician, occupational health physician. | Advisors | - Consulted | Low | Low |
| Technicians | | |  |  |  |
|  | eHealth application developer | Advisor Implementers | - Implementation partner | High | High |
|  | Technician | Support | - Co-designed - Tested prototypes | Neutral | Neutral |
|  | Medical information specialist | Advisor | - Consulted | Low | Low |
| Other experts | | | |  |  |
| Researchers | | Process facilitators | Bridging clinical, technical, and user perspectives | Neutral | Neutral |
| Policy makers, hospital managers, and insurance companies* | | Decision-makers |  | High | High |
| * In this initial phase, policymakers, hospital managers, and health insurers were identified as relevant stakeholders with decision-making roles. However, they were not actively involved in the development process, as the primary focus was on aligning the intervention with the needs and experiences of end users and care professionals. Engagement with these stakeholders is expected to become more relevant in later phases, particularly during implementation and scaling. | | | | | |

The contextual inquiry was informed by a series of complementary studies exploring the perspectives of ICU survivors and family members on digital support after critical illness. An initial national survey identified strong support for an eHealth platform providing reliable information, guidance from relevant healthcare professionals, and tailored support throughout recovery, while family members particularly emphasized the need for assistance in managing emotional distress [2]. These findings were complemented by qualitative interviews across four European countries, which highlighted experiences of powerlessness during recovery and identified key user values, including personalized information, digital consultations with healthcare professionals, peer support, and functionalities tailored to individual needs [3]. Subsequently, an international survey confirmed that perceived usefulness, accessibility, and openness towards digital health were consistent determinants of acceptance across different European countries, suggesting that these needs transcend national contexts [4]. Finally, dedicated work focusing on family members reinforced the importance of supporting relatives throughout the recovery trajectory through personalized information, professional guidance, peer support, and interventions addressing their own psychological well-being [5]. ICU nurses involved in follow-up clinics emphasized that family members often experienced the ICU admission as highly stressful, tended to downplay their own needs, and frequently struggled to understand what had happened or where to seek support after discharge [6]. Nurses’ observations underscored the importance of acknowledging emotional distress, providing clear explanations of ICU events, and offering guidance in navigating available support services. Collectively, these studies provided the evidence base for the Value Specification phase and informed the design requirements of the digital self-management intervention.

A detailed description of the scientific literature on existing eHealth applications in post-ICU recovery identified 18 completed studies and eight study protocols [7]. In brief, it addressed that digital interventions for ICU recovery primarily targeted survivors rather than family members. Interventions incorporating professional support and personalized feedback appeared more feasible and may enhance adherence. However, the findings underscored the growing potential of digital interventions to support recovery and improve quality of life among individuals with lived ICU experience.

Taken together, these contextual and literature-based insights informed several key design decisions, in particular:

1. provide clearly structured and easily accessible information,
2. allow users to engage at their own pace with availability 24/7,
3. embed components to facilitate navigation toward appropriate professional support,
4. strengthen continuity between hospital discharge and primary care follow-up.

Considerations of affordability and scalability further supported the choice for a stand-alone digital format that could be implemented broadly without continuous professional supervision.

**References**

1.	Foundation FCIC. Homepage FCIC. www.fcic.nl 2023 [cited 2023 19-09-2023].

2.	Zacharelou A, Major M, van der Meer P, van der Schaaf M, Vloet L, van Mol MM. Opinions and priorities for an e-health platform: a member consultation from an intensive care patient organisation. Australian Critical Care. 2024;37(6):882–8.

3.	Zacharelou A, Munjas Samarin R, Mikulcic K, Brcina A, Friganovic A, Jones C, et al. Value‐Based Experiences Related to Digital Follow‐Up Services Among Critical Care Survivors: An International Qualitative Study. Nursing & Health Sciences. 2025;27(2):e70135.

4.	van Olst E, Zacharelou A, Adriano F, Jones C, Nydahl P, Van Mol M. Digital Postintensive Care Support: A Comparative Survey Study Among Survivors Across Four European Countries. Journal of Patient Experience. 2026;13:23743735251414753.

5.	MMC van Mol, E. Berger, JML Latour. Value-Based Outcomes in Critical Care Recovery: The Voice of Relatives. ICU Management and Practice, 2025; 3: 133-135.

6.	Berger E, Kranenburg L, van der Veer M, Gommers D, van Dijk M, Kiers D, et al. Shaping intensive care follow-up clinics: A qualitative study of professionals’ experiences and digital integration. Intensive and Critical Care Nursing. 2026;95:104434

7.	Berger E, Schol C, Meertens-Gunput S, Kiers D, Gommers D, Rose L, et al. Digital Health Interventions Supporting Recovery for Intensive Care Patients and Their Family Members: A Scoping Review. Mayo Clinic Proceedings: Digital Health. 2025;3(1):100185
