# Supplementary material for: Participatory Development of a Digital Self-Management Intervention for Intensive Care Recovery: Multimethod Study
Source: JMIR Form Res. 2026 Nov 10;10:e99235. doi: 10.2196/99235 (PMC13750520; doi:10.2196/99235)
Supplement: Supplementary file 3 [file formative-v10-e99235-s003.docx]

Multimedia Appendix 3

Governance, technical design, and description of the intervention

Two digital self-management modules, the ICU-RecoveryPointer for ICU survivors and the ICU-FamilyCoach for family members through stakeholder engagement. Key functionalities were defined, including structured recovery information, easy 24/7 access, and guidance in navigating care.

**Project governance**

The project complied with the European General Data Protection Regulation (GDPR; EU 2016/679), the Dutch Implementation Act, and the Dutch Code of Conduct for Health Research. Data governance responsibilities and intellectual property rights were defined in a consortium agreement. The principal investigator’s institution acted as data controller. All data were pseudonymized by the research team using coded identifiers and stored on secure institutional servers. No personal data or traceable to the participants were included. Access to data was restricted to authorized research team members through password-protected systems and role-based permissions. Participants did not have direct access to the research database but could request their personal data in accordance with GDPR provisions; verified data extracts were transferred securely via encrypted file-sharing services. Data protection measures followed principles of privacy by design and security by design, including secure authentication procedures and encrypted data transfer. In the event of a suspected data breach, institutional incident response procedures were followed. The intervention was developed as a stand-alone digital application with limited integration into external clinical systems, and no cross-border data transfer agreements were required.

**Technical design**

The technical infrastructure, including hardware and software components, was implemented within an existing digital platform, [www.quli.nl](http://www.quli.nl), a Personal Health Environment (PHR, in Dutch: PGO). Therefore, the digital self-management intervention was developed and embedded within a secure app- or web-based system that enables individuals to collect and manage medical data from multiple healthcare providers in one place and to add self-measured health information. PGOs operate under the national MedMij framework, which ensures safe and standardized health data exchange.

Users access the platform through secure login procedures, including two-factor authentication. Personal health data were stored in encrypted form within a secure cloud environment, with access controls compliant with the NEN 7510 information security standard for healthcare in the Netherlands. Data storage and system management are maintained by the platform provider in accordance with applicable security standards. Data generated and stored by users within the platform remain the property of the individual user at all times.

The platform supports interoperability standards, such as Health Level Seven Fast Healthcare Interoperability Resources (HL7 FHIR), allowing for future integration with electronic health record systems. Building on this infrastructure, two digital self-management modules were developed:.

**Description of the ICU-RecoveryPointer and the ICU-FamilyCoach**

Early concepts were refined through continuous stakeholder feedback ensuring alignment with user needs and contextual feasibility until a full product was reached.

*The ICU-RecoveryPointer* supports ICU survivors by offering structured information aligned with the different phases of ICU recovery. It presents a layered structure with clickable tiles, subdivided into sections including general information, during the ICU stay, going home and the first three months after discharge, recovery beyond three months, diary, events, measurements, my files, Inforium (links to external websites), and miscellaneous resources. In addition, a simple symptom monitoring function was embedded. This tailored guidance addresses physical, psychological, and cognitive challenges after critical illness and, through self-monitoring and suggestions for support, helps users regain autonomy throughout their recovery trajectory.

*The ICU-FamilyCoach* supports family members by providing trustworthy information about ICU care, stress-monitoring features, and self-care resources to help them manage the emotional and practical burden associated with supporting an ICU patient. It presents a similar layered structure with clickable tiles covering the phases during the ICU stay and after ICU discharge. These tiles include information on, for example, the hospital environment, the ICU setting, the patient room and equipment, visiting procedures, family participation in care, and delirium. In addition, users can complete a daily stress measure that generates suggestions for coping and support.

Together, the ICU-RecoveryPointer and the ICU-FamilyCoach operationalize the core functionalities described in the main manuscript, translating stakeholder needs into practical digital features. These modules represent the first functional versions of the digital self-management intervention intended to support individuals with lived ICU experience and their family members throughout the recovery trajectory. Figure 1 shows build structure of the several layers in both modules, with content adapted to the specific target group.

Figure 1. Structured layers of the ICU-FamilyCoach and the ICU-RecoveryPointer

Logo’s used for recognizing the separate modules; the ICU-FamilyCoach [in Dutch IC-NaastenCoach] and the ICU-RecoveryPointer [in Dutch IC-HerstelWijzer].

Layered structure of the ICU-FamilyCoach, starts with five tiles within the PGO of which four are general and one tile to click for the ICU-FamilyCoach. This will subsequently provide a tile for information relevant during the ICU [Op de intensive care, the blue line] and after the ICU [Na de intensive care, the green line]. Each tile can be clicked to get one layer deeper. For example, one layer deeper in the green line, the content is divided in information on follow-up [Nazorg], about PICS and PICS-F [PICS en PICS-F], and contact with peers [Met lotgenoten]. In addition, hospital specific content is included in the next layer.

Layered structure of the ICU-RecoveryPointer shows ten tiles after the general introduction in the PGO structure. These represent General information [Algemene informatie]; Admission [Opname]; To home [Naar huis]; Recovery [Herstel]; ICU diary [IC dagboek]; Events [Gebeurtenissen]; measurements [Metingen]; and My files [Mijn bestanden]; The tiles for Information and other support means comprise content outside of the module. Each tile can be clicked to get one layer deeper. For example Admission (the green line) was subdivided in several phases in a timeline varying between general, 2 days, to discharge from the ICU. Recovery (the blue line) contains tiles with specific content with animations on the domains of PICS symptoms (physical, cognitive and psychosocial). To self-monitor these symptoms, a checklist was developed and embedded one layer deeper [Invullen van de Herstel-checklijst]. Finally, contact with peers several suggestions and websites are listed.

**Joined parties and sustainability of the intervention**

Both modules were developed in collaboration with the Foundation for Family and Patient-Centered Intensive Care (FCIC) and the patient organization IC Connect, together with national clinical and professional partners, including the Dutch nursing association (V&VN, ICU department) and the Dutch Society of Intensive Care (NVIC), as well as researchers from Erasmus MC and the University of Applied Sciences Arnhem and Nijmegen (HAN). Quli, a PGO provider, built the digital self-management intervention within its existing platform, using functionalities already applied for several target groups, for example among people with burn injuries. This joint development ensured that both digital modules provide accessible, context-specific support throughout the ICU and post-ICU recovery trajectory. Intellectual property rights are shared between FCIC, Erasmus MC, HAN, and Quli, while the final products will be owned by IC Connect after implementation.
