# Supplementary material for: Participatory Development of a Digital Self-Management Intervention for Intensive Care Recovery: Multimethod Study
Source: JMIR Form Res. 2026 Nov 10;10:e99235. doi: 10.2196/99235 (PMC13750520; doi:10.2196/99235)
Supplement: Supplementary file 4 [file formative-v10-e99235-s004.docx]

Multimedia Appendix 4

Guidance on inspirational sessions and interviews

**Topics for the inspirational sessions**

Despite promising developments, little is known about how to design and integrate eHealth, such as digital self-management interventions for ICU recovery, that adequately align with users’ needs, preferences, and the context of post-ICU recovery, particularly when professional support is not continuously available. Therefore, participatory and user-centered development approaches may offer valuable guidance for the design of a digital self-management intervention that aligns with ICU recovery contexts.

Preliminary drafts of the digital self-management intervention were developed and iteratively discussed with stakeholders to assess perceived usefulness and feasibility. The topics and relevant items, as well as existing solutions from elsewhere, guided the inspirational sessions

Summary of themes, items and existing solutions

| **Theme** | **Item – check desired functionality** | **Existing solutions** |
| --- | --- | --- |
| Chronological summary | | |
|  | Personal diary / journal feature | Digital diary  PGO* platform |
|  | Shared entries (multiple contributors) | Digital diary |
|  | Personal calendar (appointments and tasks) | Hospital web portals  PGO platform  Health apps |
|  | Progress tracking (goals and milestones) | Availability not known |
|  | Multimedia content (videos and photos) | Website IC Connect |
| Time line | | |
|  | Overview personal dashboard | Availability not known |
|  | Goal setting | Preliminary recovery app |
|  | Progress tracking | Preliminary recovery app |
|  | Milestones | Availability not known |
| Contact with peers and healthcare professionals | | |
|  | Peer support | Website IC Connect |
|  | Messaging healthcare professionals (email/chat) | PGO platform  ‘Beter Dichtbij’ (communication app) |
|  | Video calls and recorded consultations (playback available) | Hospital web portals if available |
| Digital connectedness (video calls) | | |
|  | For example:   - Bring your own device - Organization-provided devices (tablets, computers, patient TV) - Personalized content (family, children, pets) - Personal communication channels - Digital rooming-in (personal support network) - Information sharing by families and professionals | Availability not known |
| Self-monitoring and self-management | | |
|  | Questionnaires | Post-ICU clinics  PGO platform |
|  | Tracking health measures | PGO platform |
|  | Question preparation  Consultation preparation | Hospital web portals |
|  | Personal goals (patients and family members) | PGO platform |
|  | Self-management support | Availability not known |
|  | Action plan and tasks (optional guidance) | Availability not sure, explore REACH-network |
| Cognitive capacity | | |
|  | Take notice of:   - Temporarily reduced cognitive capacity and concentration - Plain language (translation of medical jargon into understandable terms) - Multiple presentation formats (visual, text, etc.) - Pacing of information delivery - Need for repetition and reinforcement | PGO platform focusing on individuals with varying levels of cognitive functioning |
| Digital (and health) literacy | | |
|  | Need for intuitive and user-friendly design | - PGO platform focusing on individuals with varying levels of cognitive functioning - Several organizations in the Netherlands such as ‘Pharos’, mental health services, geriatric services, and rehabilitation services |
|  | Physical limitations affecting technology use (strength, sensory stimuli, coordination) | Preliminary recovery app |
|  | Variation between users and recovery phases | Availability not known |
| *PGO = personal health environment | | |

**Topic list for the interviews**

Topic: Background of the aftercare nurse and aftercare clinic

- Demographic data: gender, age, number of years of work experience as an ICU nurse, number of hours per week, number of years working as an ICU aftercare nurse, average number of hours per week in aftercare, contracted hours and/or own. How much time do you schedule for an aftercare conversation (with a visit to the ICU room)?
- How much time do you additionally spend on preparation and reporting?
- Do you conduct the conversations alone, with a colleague aftercare nurse, or with an intensivist?
  - If alone or with a colleague nurse:
    - What is your experience?
    - Do you miss an intensivist in the conversation (are medical questions being asked by the patient/family)?
  - If with an intensivist:
    - What is your experience? Is the presence of an intensivist of added value? Yes/no, in what way?

Topic: Content of aftercare conversations and the (digital) support to be provided

- What topics are discussed during the conversations at the follow-up clinic?
- Do you feel you have sufficient knowledge and experience to conduct aftercare conversations? Yes/no => Why is that?
- Can you apply your knowledge and experience in the aftercare conversation? Yes/no => Can you provide an example of this?
- What is your role during these conversations (just listening or also looking at how the patient, family can be further assisted)? What can you contribute, offering in this? Is there something you feel is missing? Yes/no => What can help you with this?
- Do you also ask about the (digital) ICU diary during the aftercare conversation? Yes/no => When you think of digital tools, what else besides the (digital) diary can help the patient or relative? Have you seen examples that are applicable in ICU aftercare? Has something like this ever been mentioned in a conversation with a patient and/or relative at the aftercare clinic?
- If you ever use video calling (Teams/Zoom/Google) at the ICU follow-up clinic, what do you think about this?
- What ideas do you have that could help you in supporting the patient and/or relative? (digital and non-digital) Do you think this could or should already be offered during the patient's admission to the ICU to support the relative? (If yes, do you have ideas about this?)
- If we develop a digital intervention to provide support to the patient and relatives, what is the most important thing for you and your work activities to consider?

Topic: Authority of the aftercare nurse and ICU nurse in aftercare

*Without intensivist (perhaps with a colleague nurse?)*

- What factors within your work limit your development as a (aftercare) nurse or make it difficult for you to take on your role?
- Are there factors within your work that stimulate or assist your development as a (aftercare) nurse? Which factors are those, can you provide an example?
- If you conduct the aftercare conversation together with a colleague, do you make agreements in advance about the division of roles? (if yes, ask how, what, why)
- In what way do you share your knowledge and experience with fellow ICU nurses?
- Does an ICU nurse play a role in aftercare?
  - If yes, what role? (with patient/family?) Can this be improved?
  - If no, should this be the case? In what way?
- Could a nurse practitioner be of added value? (If yes, why? If no, why not?)
- In what way can you, as a nurse, gain and take responsibility from your role as an ICU aftercare nurse? Can you explain this in relation to your role in the nursing team?
- Do you feel that you are taken seriously by, for example, your manager or your colleagues regarding the knowledge you have about the aftercare of ICU patients, PICS, PICS-F?

*With intensivist*

- What factors within your work limit your development as a (aftercare) nurse or make it difficult for you to take on your role?
- Are there factors within your work that stimulate or assist your development as an (aftercare) nurse? What factors are those, can you provide an example?
- When you conduct the aftercare conversation together with an intensivist, do you make prior agreements about the division of roles? (if yes, ask how, what, why)
- Do you feel that you are taken seriously by the intensivist present at the aftercare conversation? => Why is that so, can you give an example?
- Do you experience that you form a team with the intensivist during the conversation? => What makes you feel that way or not, can you provide an example?
- Do you feel that you also have a say in the conversation, that you can demonstrate leadership?
- If you believe you should have more say, how do you try to achieve this?
- In what way can you as a nurse gain and take authority from your role as an ICU aftercare nurse? Can you clarify this in relation to your role in the nursing team?
- In your opinion, does an ICU nurse play a role in aftercare?
  - If yes, what role? (with patient/family?) Could this be improved?
  - If no, should this be the case? In what way?
- Could a clinical nurse practitioner be of added value? (If yes, why? If no, why not?)
- Do you feel that you are taken seriously by, for example, your manager or your colleagues regarding the knowledge you have about the aftercare of ICU patients, PICS, PICS-F?
